# EnSite NavX mapping system guided implantation of a dual-chamber permanent pacemaker in a 41-year-old pregnant woman with a 4-year follow-up

**DOI:** 10.1186/s12872-022-02764-w

**Published:** 2022-07-21

**Authors:** Peng Wang, Guang-Sheng Wei, Jun-Hua Wang, Yan-Jie Cao, Wei-Wei Zhu, Hang Shen, Zhi-Yue Zhang, Li Ai, Meng Wang

**Affiliations:** 1 Department of Cardiology, Air Force Medical Center, Air Force Medical University, Beijing, 100142 China; 2 Graduate School of Hebei North University, Zhangjiakou, 075000 Hebei China; 3 Department of Internal Medicine Teaching Research, Air Force Medical Center, Air Force Medical University, Beijing, 100142 China; 4 Abbott Medical (Shanghai) Co. Ltd., Beijing, 100028 China

**Keywords:** EnSite NavX mapping system, Fluoroscopy, Permanent pacemaker implantation, Lead displacement

## Abstract

**Background:**

X-ray fluoroscopy has been the primary cardiac imaging modality in permanent pacemaker implantation (PPI) operations, but it inevitably results in radiation exposure for both operators and patients. Fluoroscopy is considered a contraindication, especially in certain circumstances, such as gestation, during which the fetus is most sensitive to radiation exposure. Therefore, measures to avoid radiation exposure are necessary, and a more safe and feasible approach is needed for this procedure. Since the EnSite NavX mapping system (ENMS) can create the required geometric contours of those relevant cardiac structures and chambers, it can be used as an alternative to X-ray fluoroscopy in PPI. In addition, because the displacement of atrial leads is a common complication of PPI, lead displacement may occur more readily without fluoroscopic guidance. Therefore, reliable measures are required to prevent leads from displacement.

**Case introduction:**

A 41-year-old woman at the 15th week of gestation was referred to our department with recurrent episodes of syncope and amaurosis fugax for 2 years. Holter monitoring showed sinus rhythm, Mobitz Type II atrioventricular block and high-grade atrioventricular block with ventricular arrest up to 4945 ms. A dual-chamber PPI was performed successfully for the patient under the guidance of the ENMS instead of fluoroscopy. Displacement of atrial lead was effectively avoided by bending the top of atrial lead before implantation and making it a U-shape during operation, which left space for possible subsequent external pulling stress.

**Conclusions:**

For PPI, ENMS is a feasible and reliable alternative to traditional X-ray fluoroscopy, especially when performing operations on pregnant patients. By bending the top of the active-fixation atrial lead into a U-shape during operation, the displacement of atrial lead may be avoided.

## Background

X-ray fluoroscopy is routinely used for cardiac imaging in permanent pacemaker implantation (PPI) operations. However, fluoroscopy is considered a contraindication that should be avoided in some special cases, such as pregnant women and children. Therefore, other reliable alternatives are desired. As the EnSite NavX mapping system (ENMS) can create the required geometric contours of those relevant cardiac structures and chambers, it can be used as a replacement for X-ray fluoroscopy in PPI [1]. Additionally, since the displacement of atrial leads is a common complication after PPI operations, lead displacement may tend to occur more easily without fluoroscopic guidance. Thus, reliable measures are required to prevent leads from displacement. This report presents a successful operation of PPI guided by ENMS for a 41-year-old pregnant woman with 4-month-old twins. Effective techniques are applied to prevent the atrial lead from displacement in this case.

## Case presentation

**Fig. 1:**
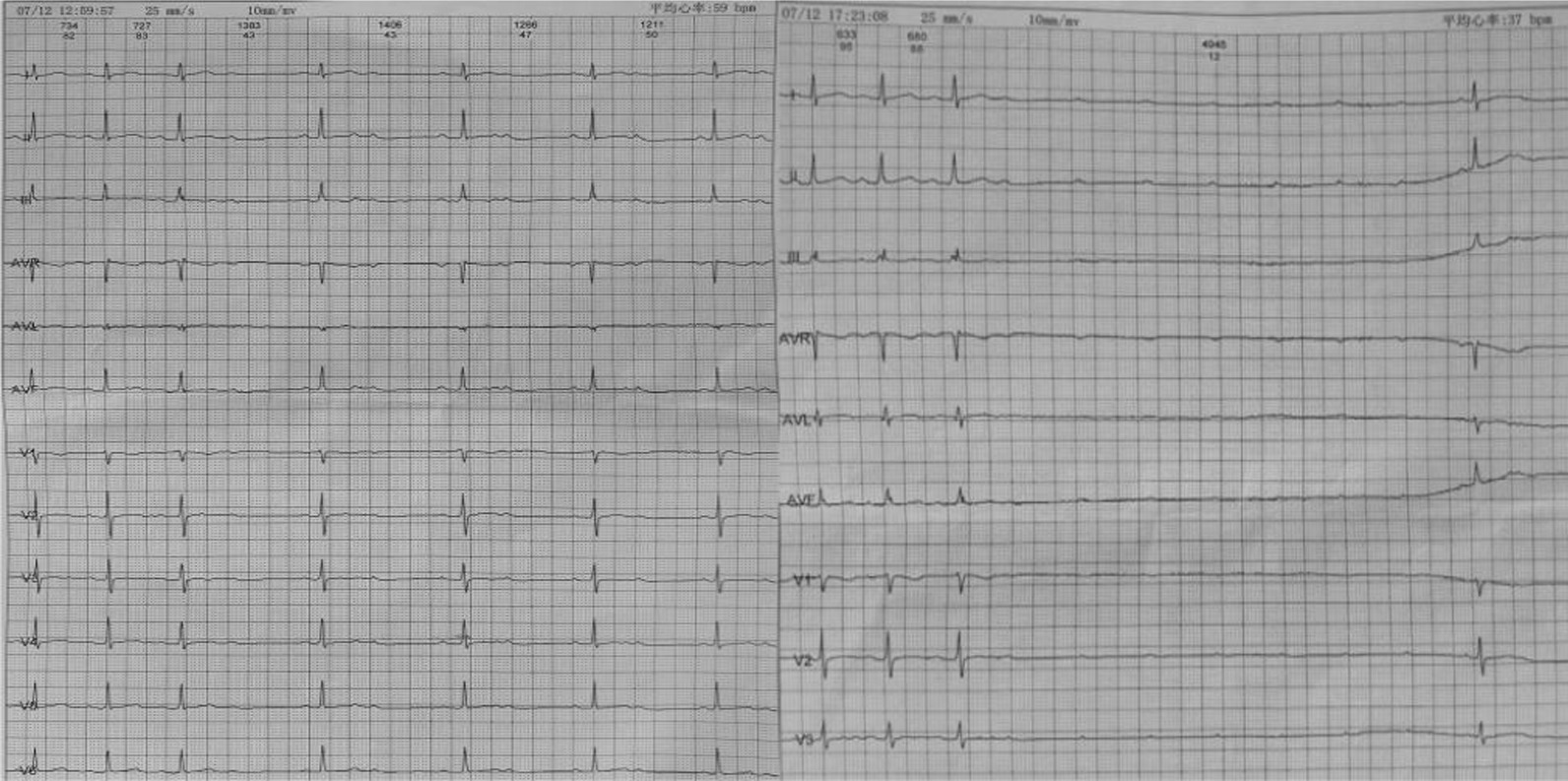
A 24-h Holter monitoring shows Mobitz Type II atrioventricular block (left) and high-grade atrioventricular block with ventricular arrest up to 4945 ms (right) associated with syncope

A 41-year-old pregnant woman at the 15th week of gestation with recurrent episodes of syncope and amaurosis fugax for 2 years was referred to our department on July 16, 2017. Physical examination: blood pressure 118/85 mmHg; heart rate 58 bpm, irregular, no cardiac murmurs. The 24-h Holter monitoring showed sinus rhythm, Mobitz Type II atrioventricular block (Fig. 1, left), high-grade atrioventricular block with ventricular arrest up to 4945 ms (Fig. 1, right) when the patient suffered from amaurosis fugax. Echocardiographic examination showed negative. Serum creatine kinase (CK) 33u/l, creatine kinase MB subfraction (CK-MB) 11u/l, and other laboratory results were also negative. The patient had no hypertension, cardiomyopathy or other cardiovascular diseases. She had no any family history of genetic diseases either. Therefore, acute myocardial injury was excluded as the cause of the atrioventricular block. Finally, a dual-chamber PPI was performed under the guidance of ENMS to prevent the pregnant woman and the fetus from radiation exposure.

**Fig. 2:**
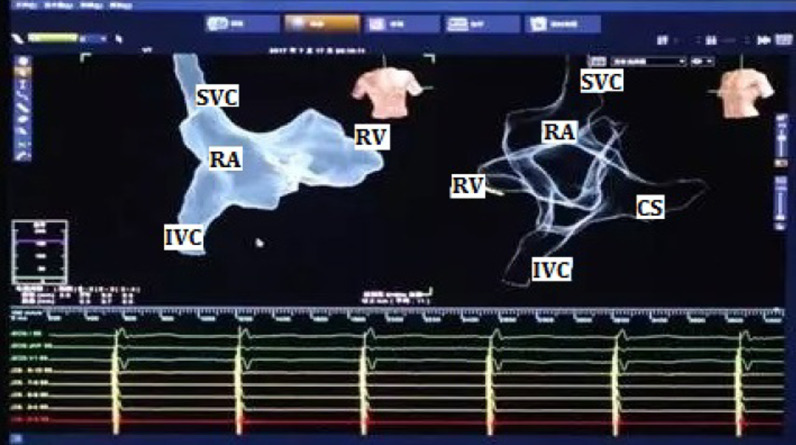
The three-dimensional anatomical map of heart chambers drawn by ENMS. SVC, superior vena cava; IVC, inferior vena cava; RA, right atrium; RV, right ventricle; CS, coronary sinus

**Fig. 3:**
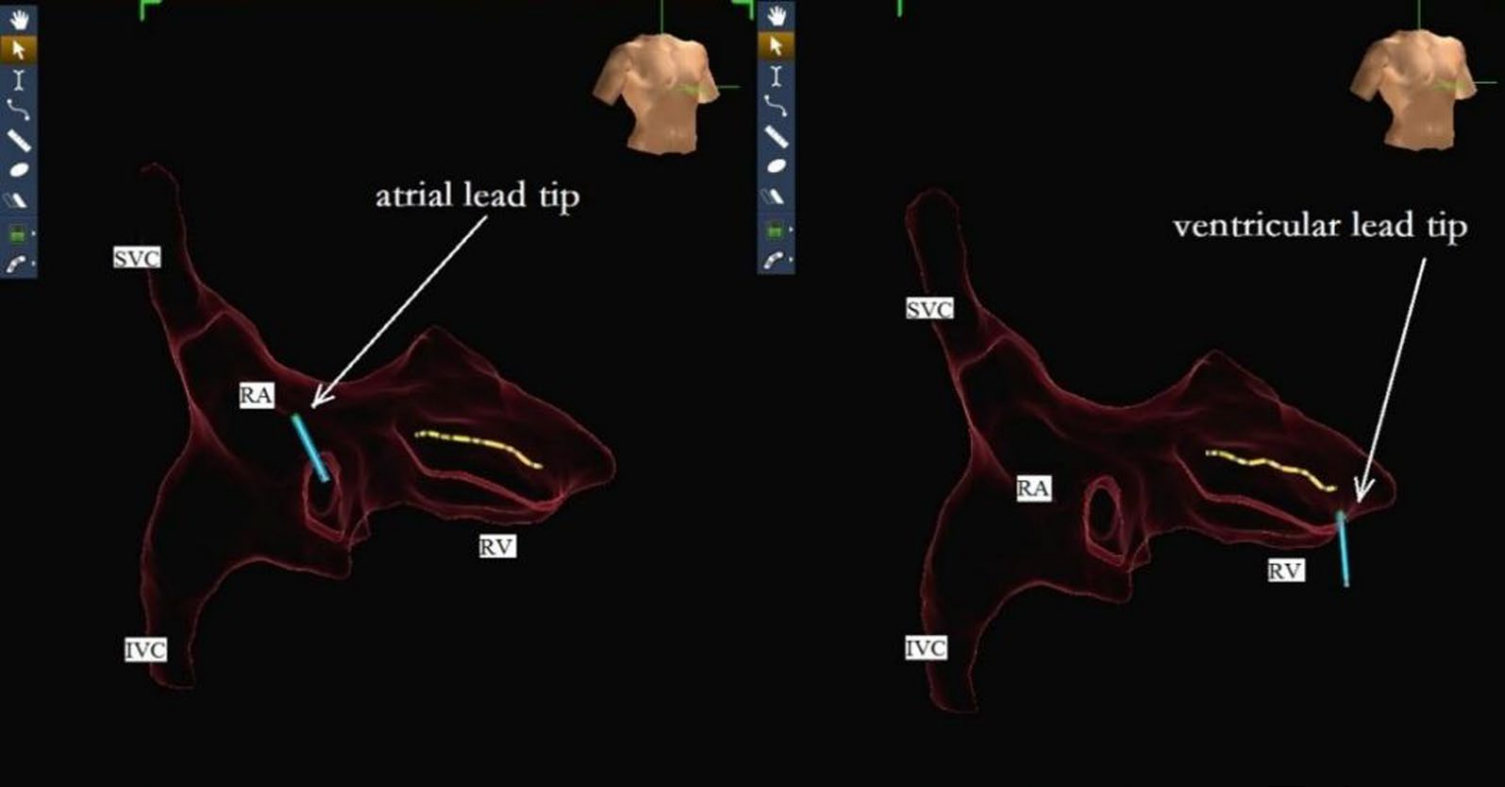
The right anterior oblique view of the patient shows the positions of the two leads. SVC, superior vena cava; IVC, inferior vena cava; RA, right atrium; RV, right ventricle

**Fig. 4:**
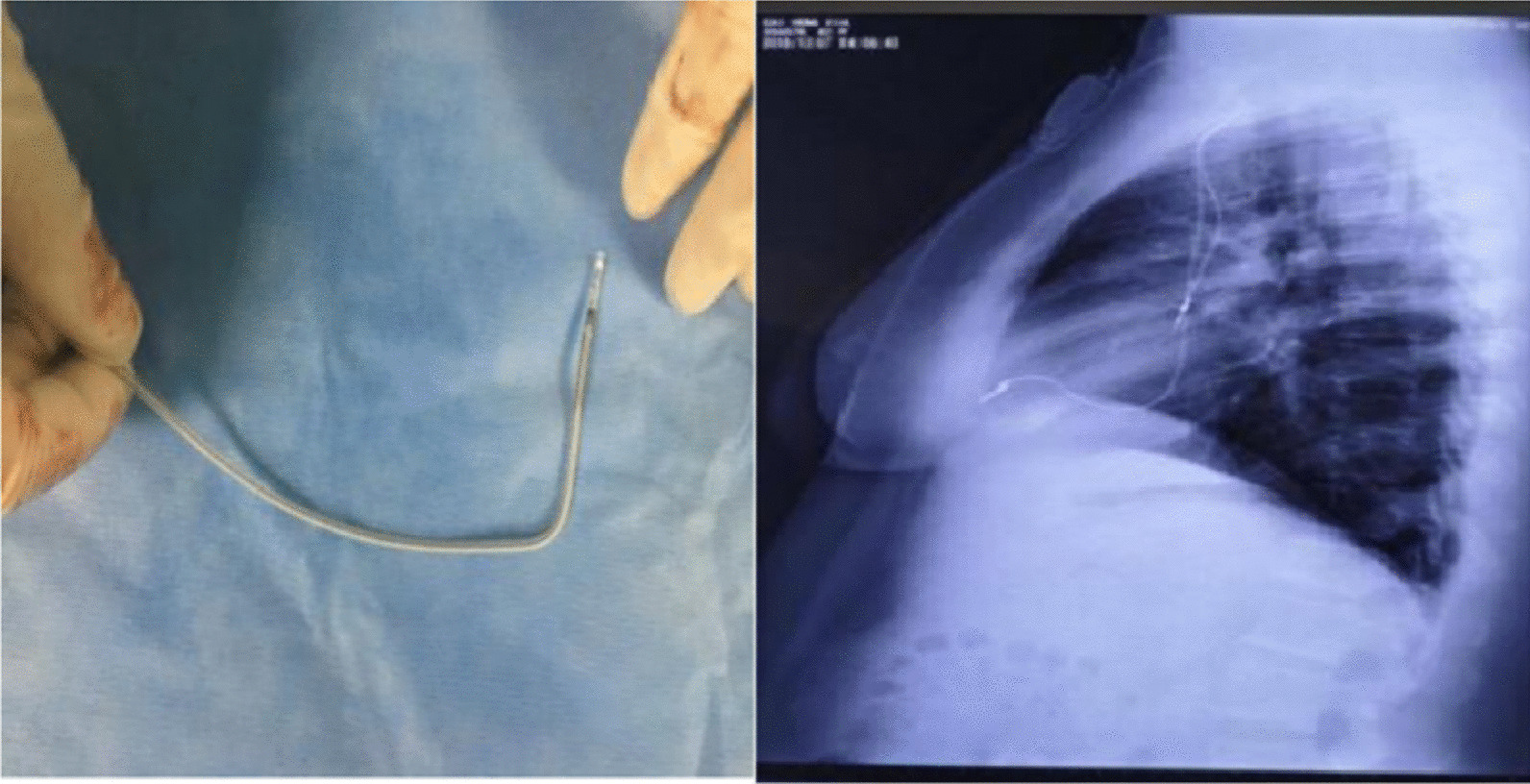
Atrial lead with a bigger bend before implantation (left). X-ray shows the two positions of leads 5 months after cesarean section (right)

The operation was performed on July 18, 2017. The method of ENMS for drawing the three-dimensional anatomical map of heart chambers was identical to that in the radiofrequency ablation operation. First, three pairs of mutually perpendicular electrode pads were attached around the patient's heart to form a stable special electric field. In this way, when a metal electrode catheter with microcurrent connected to ENMS was placed in this electric field, its precise three-dimensional position could be displayed on the screen. Then a 7F sheath was inserted into the right femoral vein under local anesthesia. After connecting an alligator cable to the ENMS, a 10-polar catheter was inserted into the sheath and advanced to the inferior and superior vena cava, right atrium, tricuspid annulus, and right ventricle to create the geometry contours of those chambers by wagging and moving it inside those cavities (Fig. 2). Another 7F sheath was inserted into the left subclavian vein through a percutaneous puncture. Under the guidance of ENMS, the active-fixation leads of the right atrium and right ventricle were manipulated and secured to proper positions (Fig. 3). Based on our previous experience, to avoid the displacement of atrial leads, the front of the atrial lead was bent into a curved shape before implantation (Fig. 4, left). The atrial lead was continued to be pushed forward by about 5 cm after implantation in the proper position under transthoracic echocardiography guidance to make the front of the atrial lead a U-shape. The parameters were tested, as shown in Table 1. Then the leads were connected to the pacemaker in DDD mode, and the pacemaker was implanted into the left infraclavicular subcutaneous pocket. The total operation time was 68 min. The patient was discharged and followed up regularly. Boy and girl twins were successfully delivered by caesarean section 4 months after the pacemaker operation. In the 5th month after the operation, though the curvature of atrial leads became smaller than its original curvature (Fig. 4, right) and the sensed amplitude of atrial leads decreased, other parameters, such as the pacing thresholds and impedance of the two leads, were normal (Table 1). The patient had no relevant unpleasant symptoms. The two leads were regularly tested for 4 years without any intervention.

**Table 1:** Lead parameters in different periods

	Impedance (Ohm)		Threshold (V@0.4 ms)		Sensed amplitude (mV)	
	Atrial	Ventricle	Atrial	Ventricle	Atrial	Ventricle
Jul.18, 2017	650	550	0.6	0.5	1.5	14.0
Aug.25,2017	943	554	0.5	0.5	1.4	15.2
Dec.13, 2017	884	515	0.5	0.5	0.7	14.6
Aug.17, 2019	872	462	0.5	0.5	0.5	14.2
Nov.27, 2021	828	446	0.5	0.5	0.4	15.6

## Discussion

Fluoroscopy is the primary cardiac imaging method in PPI operations, but it inevitably exposes operators and patients to radiation. Fluoroscopy is considered a contraindication for pregnant women, especially those between the 8th to 15th week of pregnancy, when the fetus is most sensitive to radiation. Thus, a more feasible cardiac imaging approach is needed instead of fluoroscopy. Currently, in international medical guidelines, no methodological recommendations are available on how to avoid or reduce radiation exposure for PPI in pregnant women. In some reported cases [1, 2], transthoracic or transesophageal or even intracardiac echocardiography was used to guide the implantation of pacemaker leads. However, ultrasound is not widely used for cardiac imaging in PPI due to the visualization problems of all leads or complications such as aspiration pneumonia or suffocation [3].

The ENMS is based on the measurement of electrical currents for the three-dimensional localization of catheters. A low-amplitude, high-frequency electrical current was delivered through three pairs of skin patches located on the patient's chest in orthogonal planes, creating a voltage gradient along each direction and acquiring geometric contours of those relevant cardiac structures. At present, ENMS has been used in radiofrequency ablation treating cardiac arrhythmia, such as atrial fibrillation and premature ventricular contraction. Ping Guo et al. [3] reported six cases of PPI with zero-fluoroscopy under the guidance of ENMS, including one pregnant woman. No complications were observed during 6 months of follow-up. An Italy multicentre registry study during 2011–2014 [4] on cardiac resynchronization therapy (CRT) device implantation concluded that ENMS-guided CRT implantation was proven safe and effective in both high- and low-experienced centers and could reduce fluoroscopy. Clinical studies suggested that ENMS may become the trend for cardiac imaging to guide PPI instead of fluoroscopy to reduce radiation exposure.

Displacement of atrial leads is a common complication after PPI operations, and late displacement is defined as the displacement occurring more than 6 weeks after pacing system implantation [5, 6]. Generally, after PPI, the upper body and respiratory movement of the patient will inevitably cause a certain degree of pulling force on the pacing leads, which may lead to lead displacement and a significant increase in the pacing threshold. In particular, displacement of pacing leads during delivery could be caused by many factors, such as deep breathing in the follow-up delivery process, large-scale changes in body posture, cesarean section, and mechanical ventilation used for anesthesia. Therefore, an effective technique was adopted in this case to prevent lead displacement. In this way, some space could be left for possible external pulling stress after the operation. Even if the U-shaped arc was straightened, the tip of atrial lead would not be dislocated, thereby ensuring the stability of the pacing threshold. According to the post operative follow-up, this technique successfully prevented lead from displacement. The threshold value did not change in the follow-up after the cesarean section. However, the sensing P-wave lightly decreased, possibly because the U-shaped arc of the atrial lead was straightened to some extent, which was confirmed by subsequent X-ray fluoroscopy examination. As the lead tip did not move, the stability of other parameters, such as pacing threshold and impedance, and the normal pacing function were guaranteed.

## Conclusion

This study revealed that ENMS is a feasible and reliable alternative to traditional X-ray fluoroscopy in PPI operations, especially performing such operations on pregnant patients. Displacement of atrial lead can be avoided by bending the front part of the active-fixation lead of the right atrium into a U-shape in PPI operation.

## Data Availability

The datasets used and analyzed in the present report are available from the corresponding author on reasonable request.

## Abbreviations

PPI: Permanent pacemaker implantation
ENMS: EnSite NavX mapping system
LVEF: Left ventricular ejection fraction
CRT: Resynchronization therapy
